# Optimization of Single α-Phase for Promoting Ferromagnetic Properties of 44Fe–28Cr–22Co–3Mo–1Ti–2V Permanent Magnet with Varying Co Concentration for Energy Storage

**DOI:** 10.3390/ma15072344

**Published:** 2022-03-22

**Authors:** Inam Ullah Khan, Vineet Tirth, Ali Algahtani, Rajwali Khan, Mohammad Sohail, Amjad Ali, Saiful Islam, Kashif Irshad

**Affiliations:** 1 Department of Physics, COMSATS University Islamabad (CUI), Islamabad 44000, Pakistan; inam_mwt@yahoo.com; 2 Mechanical Engineering Department, College of Engineering, King Khalid University, Abha 61421, Saudi Arabia or v.tirth@gmail.com (V.T.); alialgahtani@kku.edu.sa (A.A.); 3 Research Center for Advanced Materials Science (RCAMS), King Khalid University, P.O. Box No. 9004, Guraiger, Abha 61413, Saudi Arabia; 4 Department of Physics, University of Lakki Marwat, Lakki Marwat 28420, Pakistan; msohail@ulm.edu.pk; 5 Interdisciplinary Research Center for Renewable Energy and Power Systems (IRC-REPS), King Fahd University of Petroleum & Minerals, Dhahran 31261, Saudi Arabia; kashif.irshad@kfupm.edu.sa; 6 Civil Engineering Department, College of Engineering, King Khalid University, Abha 61421, Saudi Arabia; sfakrul@kku.edu.sa; 7 K.A.CARE Energy Research & Innovation Center at Dhahran, Dhahran 31261, Saudi Arabia

**Keywords:** thermomagnetic treatment, magnetic properties, phase analysis, transformation, structural properties, morphology

## Abstract

The thermal stability and structural, microstructural and magnetic properties of (40 + x) Fe–28Cr–(26 − x) Co–3Mo–1Ti–2V magnets with x = 0, 2, 4 addition in cobalt content were investigated and presented. The magnetic alloys were synthesized by vacuum arc melting and casting technique in a controlled argon atmosphere. Magnetic properties in the alloys were convinced by single-step isothermal field treatment and subsequent aging. The alloys were investigated for thermal stability, structural, microstructural and magnetic properties via differential thermal analysis (DTA), X-ray diffractometery (XRD), optical microscopy (OM), field emission scanning electron microscope (FESEM) and DC magnetometer. Metallurgical grains of size 300 ± 10 μm were produced in the specimens by casting and refined by subsequent thermal treatments. The magnetic properties of the alloys were achieved by refining the microstructure, the optimization of conventional thermomagnetic treatment to modified single-step isothermal field treatment and subsequent aging. The best magnetic properties achieved for the alloy 44Fe–28Cr–22Co–3Mo–0.9Ti–2V was coercivity H_c_ = 890 Oe (71 kA/m), B_r_ = 8.43 kG (843 mT) and maximum energy product (BH)_max_ = 3 MGOe (24 kJ/m^3^). The enhancement of remanence and coercivity enabled by the isothermal field treatment was due to the elongation of the ferromagnetic phase and step aging treatment due to the increase in the volume fraction. This work is interesting for spin-based electronics to be used for energy storage devices.

## 1. Introduction

Fe–Cr–Co alloy-based permanent magnets such as Rare-earth, Alnico, Ceramic Ferrites with both low and high temperature magnetic properties are known for their superior ductility, high corrosion resistance and budget-dependent applications in modern industry from electronic chip and magnetic disks to huge generators [1]. Alnico, Fe–Cr–Co, and Sm–Co permanent magnets in particular have high temperature capabilities with varying ductility, strength, and corrosion. To satisfy the needs of contemporary industry, the development of specific machinery in aviation, shipbuilding, and high-end technology necessitates the use of materials with excellent mechanical, magnetic, and temperature/corrosion resistance capabilities. H. Kaneko et al. reported a permanent magnetic alloy based on Fe–Cr–Co in 1972 [2]; later, qualities were strengthened by compositional alteration (such as Mo, Ti, V, Si, W, Zr, Al, B, and Sn, for example) or process optimization, which was deferred owing to intensive Alnico research beginning in the 1930s. Fe–Cr–Co alloys offer great ductility, strong corrosion resistance, cheap cost with regard to Co and Sm, and significantly better magnetic characteristics compared to Alnico-5 and 8. These alloys have the same metallurgy and magnetic characteristics as Al–Ni–Co magnets. Fe–Cr–Co magnetic alloys comprise 2–25 in wt.% Co and 20–35 in wt.% Cr with Fe balance [2,3,4]. Transmission electron microscopy investigations have revealed that the pinning of domain walls is the principal mechanism of coercivity in Fe–Cr–Co alloys. The key benefits of Fe–Cr–Co magnets are their large Curie temperature (over 600 °C), functioning temperature (up to 500 °C) and remanence that is equivalent to A–Ni–Co magnets but superior to typical Cr-steel, Co-steel, Ba-ferrites, Cunico and Cunife magnets [5]. Another procedure utilized in the manufacture of Fe–Cr–Co magnets [5] included solution treatment (ST) at 1200–1300 °C in argon, followed by quick quenching, isothermal field treatment (IFT) and step aging treatments without magnetic field at 600–560 °C for (6–20) h. The best magnetic properties obtained in Fe–Cr–Co magnets are H_c_ = 580 Oe (46 kA/m), B_r_ = 13 kG (1300 mT) and magnetic density (BH)_max_ = 5.4 MGOe (43 kJ/m^3^). The Fe–Cr–Co system alloys have a ferromagnetic (FM) bcc α-phase structure in the temperature range of 1200–1300 °C. At moderate temperatures (10,500 °C), the magnetic phase coexists with the nonmagnetic phase (δ-phase). At elevated Cr concentrations, a brittle, nonmagnetic phase emerges in this temperature range as well. Among three phases, only the α-phase is FM. It can be retained at room temperature if the samples are quenched from the high temperature (1200–1300 °C). Aging in the temperature range of 620–700 °C (depending upon the composition of the alloy) decomposes the α-phase spinodally into highly FM (Fe, Co-rich) α_1_-phase (rod-like particles) and a paramagnetic (Cr-rich) α_2_-phase, both of which maintain a bcc structure. The spinodal decomposition temperature is approximately 550 °C for the binary 30%Cr–Fe alloy and increases to 650 °C for the ternary 20%Co–30% Cr–Fe alloy. Hence, cobalt is desirable since it increases the spinodal decomposition temperature [6,7]. On the other hand, cobalt is a γ-phase former, which is undesirable. In general, as the cobalt content increases, the coercivity and energy product increases while the remanence slightly decreases.

Additionally, the alloys have the best magnetic characteristics when they are composed of an aligned, extended, single-domain nanocrystalline precipitate of the highly magnetic α_1_ phase regularly scattered in a weakly magnetic 2 matrix [8]. When a high-temperature composition of the alloy with a homogenous single-phase structure is brought inside the spinodal range at a lower temperature, it transforms into a separated two-phase structure, a process known as “spinodal decomposition.” The dissolved and decomposed alloy has a periodic microstructure in the order of hundreds of angstroms and is formed of two isomorphous phases, one of which is in the form of a small precipitate evenly dispersed in another phase that forms the matrix [9]. Isothermal field treatment (IFT) is critical for improving the permanent magnetic characteristics of Fe–Cr–Co and Alnico alloys. These two types of alloys experience a single-phase bcc structure solid solution spinodally decomposed into two isomorphic phases, one of them is FM and the other is paramagnetic [10]. The FM phase below Curie temperature are dominant and the FM phase above Curie temperature is a strong paramagnetic region. The Curie temperature of steel magnets (for example, Alnico, Fe–Cr–Co, etc.) and rare-earth magnet (for example, SmCo_5_) is in the range of 650–860 °C. The magnetic field is externally employed during the isothermal treatment to elongate the ferromagnetic phase and thus impart magnetic anisotropy to these types of alloys [11]. The microstructure appears to be anisotropic, in which FM Fe and Co-rich particles were elongated in the direction of the applied field, usually in the <100> or <110> directions [12]. After thermo-magnetic treatment, the samples are aged. During the aging treatment, the samples are heated at 610 °C for 3 h and then cooled at the rate of 25 °C/h to 490 °C and held for 5 h after which samples are furnace cooled. The optimum magnetic properties achieved to date by magnetic field aging in 52Fe–30Cr–15Co–3Mo alloys are H_c_ = 1025 Oe (82 kA/m), B_r_ = 11.5 kG (1.15 T), (BH)_max_ = 7 MGOe (56 kJ/m^3^). Deformation aging is a technique of achieving magnetic anisotropy in an elongated precipitate structure through plastic deformation rather than magnetic field treatment. The alloy is first cooled through the spinodal temperature to develop oversized, nearly spherical particles in deformation aging. An anisotropic deformation such as an extrusion wire then drawing or rolling the alloys to elongate and align these spherical particles. The optimum magnetic properties achieved to date by a deformation aging process are H_c_ = 1080 Oe (86 kA/m), B_r_ = 13 kG (1.30 T), (BH)_max_ = 9.8 MGOe (78 kJ/m^3^) for 42Fe–33Cr–23Co–2Cu alloy [13,14]. The deformation aging technique obtains the magnets in a thin sheet or wire shape. Deformation aging produced thin magnetic sheets, rods or wires which produces maximum magnetic properties as H_c_ = 85.8 kA/m (1080 Oe), Br = 1.3 T (13 kG) and (BH)_max_ = 79 kJ/m^3^ (9.8 MGOe) in (42Fe–33Cr–23Co–2Cu) alloy [15], but these are expensive due to the involvement of a long processing cycle which put a restriction on their practical applications.

In this paper, we studied the effect of Co concentration on the thermal stability and the structural, microstructural and magnetic properties of Fe–Cr–Co–Mo system alloys with the microalloying of Ti and V metals. In the present work, anisotropic Fe–Cr–Co–Mo-Ti–V-based permanent magnetic alloys were produced by casting technique. The magnetic properties of the alloys were achieved by refining the microstructure, optimizing the conventional thermomagnetic treatment to modified single-step isothermal field treatment and subsequent aging. The best magnetic properties achieved for the alloy 44Fe–28Cr–22Co–3Mo–0.9Ti–2V included the coercivity H_c_ = 890 Oe (71 kA/m), B_r_ = 8.43 kG (843 mT) and maximum energy product (BH)_max_ = 3 MGOe (24 kJ/m^3^). The enhancement of remanence and coercivity enabled during the isothermal field treatment was due to the elongation of the ferromagnetic phase and the step aging treatment due to the increase in the volume fraction. The goal of this invention was to provide an improved alloy system with excellent magnetic properties, particularly high magnetic hardness (i.e., rententivity or coercive force) and a maximum energy product, while remaining low in material cost and workability and only requiring a relatively simple manufacturing procedure.

## 2. Materials and Methods

Specimen ingots using a raw material purity of 99.9% and a weight (30–35 g) of nominal composition (in wt.%), alloy Sample A (40Fe–28Cr–26Co–3Mo–0.9Ti–2V), alloy Sample B (42Fe–28Cr–24Co–3Mo–0.9Ti–2V) and alloy Sample C (44Fe–28Cr–22Co–3Mo–0.9Ti–2V) were prepared by vacuum arc melting furnace under an Ar inert atmosphere. Rectangular bricks of volume 20 × 18 × 8 mm^3^ were obtained from the casted ingots to facilitate the processing parameters and characterization methods of bulk samples. The processing parameters consist of solution treatment (ST), isothermal field treatment (IFT) and subsequent aging at predetermined cooling rates. The conventional solution treatment used for the present work is 1250 °C for the stay time of six hours followed by compressed blowing air quenching from 1250 °C to room temperature.

**Figure 1:**
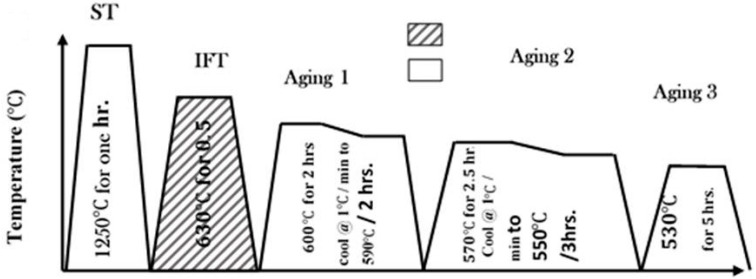
The processing flow chart for the alloys (40 + x)Fe–28Cr–(26 − x)Co–3Mo–1Ti–2V.

In order to find the optimum condition of the heat treatment for the ridge alloys, the optimum isothermal field treatment was first schematically determined by the procedure shown in Figure 1. It should be noted that the specimens are not continuously slow-cooled to the IFT temperature because of the formation of a γ or δ phase during the cooling. The IFT applied to the considered alloys was 620–640 °C for 10–35 min followed by slow cooling to 600 °C at the rate of 1 °C per minute under external magnetic field and then air cooled. The intensity of the external magnetic field was 4.86 kOe (400 kA/m) for the specimen throughout the present work. The optimized temperature for IFT was 630 °C and the optimized soaking time was 30 min followed by 1 °C per minute, cooling to 600 °C and then specimen bricks were subjected to subsequent aging treatments of Age 1, Age 2 and Age 3.

The chemical composition of the specimen verified by wet chemical method after casting through X-ray fluorescence (XRF) and energy-dispersive X-ray spectrometer (EDX) and the average results of compositional analyzers are presented in Table 1. The small amounts of carbon, sulfur and nitrogen in ppm found in the specimen ingots are the proof of the materials’ purity.

Specimens were prepared for the microstructural examination by microcutting, forcipol grinding and polishing, followed by electrolytical etching in 10% chromic acid for 20 s and 4 volts. The microstructural properties were observed by Olympus (BX 61) optical microscope (OM) and the spinodal structure examined by FEI (Quanta 450) scanning electron microscope (FESEM). Phase analysis was carried out by X-ray diffraction (XRD) with CuKα (1.54 Å) radiation after calibration with a standard sample at room temperature. Thermal stability was analyzed after complete treatment by STA 449F3 (NETZSCH, Selb, Germany) for differential thermal analyses (DTA) under the Ar inert atmosphere. The inert atmosphere of argon was applied through protective and purge nozzles at the combined rate of 70 mL/min at temperatures from 450 °C to 850 °C at 5 °C/min. The analyses of the Sample A, Sample B and Sample C specimens were performed after flushing two times with the vacuum which attained thermal equilibrium conditions. The baseline was created for the specimen under the same conditions with an empty reference and the sample crucible with the lid at the given specified temperature range with the same heating and inert conditions was used for the Sample A, Sample B and Sample C alloys. The calibration files for temperature and sensitivity were created with the pure material of the melting point ranges from room temperature to 1400 °C. Magnetic properties were measured by DC magnetometer after calibrating with standard samples at room temperature.

## 3. Results and Discussion

**Figure 2:**
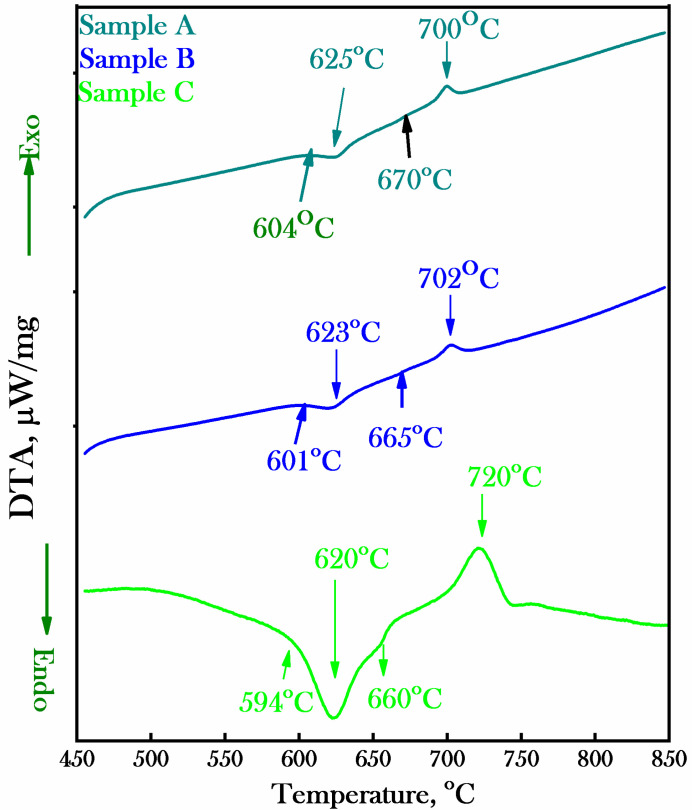
Differential thermal analysis versus temperature of Sample A, Sample B and Sample °C alloys after the complete heat treatment showing the onset, peak and phase transformation temperatures.

The results of DTA as a function of temperature for the presented Sample A, Sample B and Sample C alloys after complete processing are shown in Figure 2. Calibration files used for correction, temperature and sensitivity were 5 °C per min with an argon inert atmosphere in a silicon carbide heating element furnace. The silicon carbide heating element furnace has capability of ±1 °C accuracy with a controlled heating rate of 1–50 °C/min (RT-1600) °C. The Sample A, Sample B and Sample C alloys were analyzed for the temperature range of 450–850 °C in the controlled Ar inert atmosphere at the heating rate 5 °C/min as well as for the baseline. The alloys were analyzed with baseline correction file prepared by empty reference and the sample crucibles plus lid from room temperature (RT) to 1200 °C at the rate of 5 K/min in the Al_2_O_3_ crucible and lid. The weighing conditions for the sample crucible plus lid and reference crucible plus lid were 30 mg and 1456.8 mg, respectively. The inert argon gas flow at protective and purging was 20 and 50 mL/min.

The thermal gravimetry analysis curve shows almost zero weight loss with heating due to the bulk shape of the hard magnetic material for all samples. The DTA curve shows endo- and exo-peaks for each investigated alloy with appreciable difference in the temperature of the two peaks which is responsible for α_1_ and α_2_ phases. The temperature peaks were 604 °C, 625 °C, 670 °C and 700 °C for the Sample A alloy; 601 °C, 623 °C, 665 °C and 702 °C for the Sample B alloy; and 594 °C, 620 °C, 660 °C and 720 °C for the Sample C alloy. The onsets for the endothermic and Curie temperature were 604 °C and 670 °C for Sample A; 601 °C and 665 °C for Sample B; and 594 °C and 660 °C for Sample C, respectively. The difference in the temperature of the peaks was 75 °C for the Sample A alloy, 79 °C for the Sample B alloy and 100 °C for the Sample C alloy. The difference in the peaks temperature and height of each peak increases with the decrease in Co content. Thus, the cobalt content considered the γ- and δ- phase enhancer and stabilizer for the presented alloy system of the Fe–Cr–Co–Mo family. The difference in peaks temperature and height related to the difference between the α_1_ and α_2_ phases may be directly related to the magnetic properties of alloys. The specimens of the Sample A alloy, the Sample B alloy and the Sample C alloy were analyzed at same optimized thermal treatment cycle shown in Figure 1 and with a slow heating rate (5 °C/min) during the thermal analysis to study the magnetic properties.

### 3.1. X-ray Diffraction Studies

**Figure 3:**
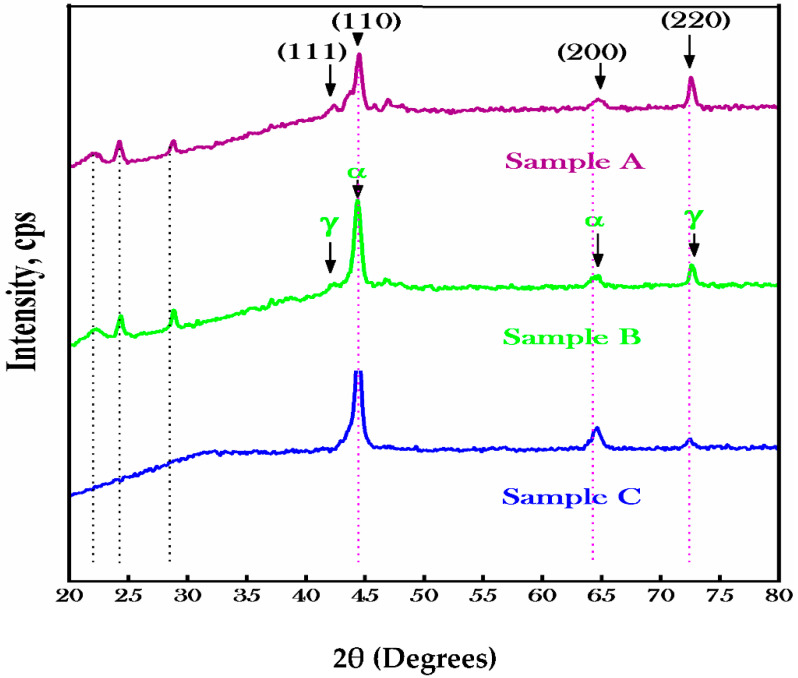
The X-ray diffraction pattern of the Sample A alloy, Sample B alloy and Sample C alloy after complete heat treatment showing the single solid solution of the α-phase accompanied by some unwanted γ-phase.

The X-ray diffractometry of the investigated specimens of Sample A, Sample B and Sample C after the complete heat treatment was presented in Figure 3. The Cukα radiation of wavelength 1.54 Å with a step size of 0.02 in the range of 10–80 of 2θ° Bragg’s angle were used for the structural study of the alloys. It was shown that peak broadening occurred at 21.9°, 24.09°, 28.7°, 42.08°, 44.4°, 47.1°, 64.7° and 72.4° in the Sample A alloy; at 22.1° 24.43°, 28.89°, 42.1°, 44.41°, 65.05° and 72.63° in the Sample B alloy and at 44.4°, 64.5° and 72.44° in the Sample C alloy. The accuracy of determining the Bragg’s angle was ±2°, while the peak broadening in the Sample C alloy corresponds to 31° with a crystal plane (100) and bcc structure related to the α-phase. Angle 2θ° at 22, 24.5, 29, 43 and 72.5 relates to the (111) and (220) crystal planes in Sample A alloy and Sample B alloy having an fcc crystal structure with γ- and δ- phases composition rich in Fe and Cr(M) where (M = Mo, Ti, etc.).

The unwanted γ- and δ-phases enhance the decomposition at high temperature but weaken the magnetic properties in an insolent way. It was noted that the peaks broadening corresponds to angles of 44.5 and 65 with a maximum peak intensity from 3000 to 4500 cps related to the (110) and (200) crystal plane with a bcc structure. The bcc crystal structure corresponds to (100), (110) and (200) crystal planes whose existence proves the single α-phase solid solution material which is in agreement with the literature [16,17,18]. The bcc structure at the ridge regime temperature from 620 to 645 °C decomposes into two isomorphic phases α_1_ and α_2_ with a composition of Fe–Co and Cr–Mo. The shoulder of the high intensity peaks was the symbol of spinodal decomposition of the single-phase solid solution, i.e., with a small lattice parameter difference between the two sublattices. Regarding treatment, no change occurred in the composition but due to diffusion, the composition adjustment occurred which led to a decrease in the gamma phase. Fast quenching produced the indissoluble isolated metastable phase by γ-phase and in the aging process which accelerated the decomposition of the α-phase, leading to the complete disappearance of or reduction in intensity as for the Sample C alloy. During the heat treatment of the specimen, a little shift of 0.3° occured for the crystal plane (110) which caused heterogeneous homogenization leading to the production of internal stresses in the alloys. The growth of the preferred crystal plane (110) of the Sample C alloy was the confirmation of good and the multiple unwanted crystal planes in the Sample A alloy and Sample B alloy were related to the reduction in magnetic properties. By increasing the Co content, allotropic phases of Fe in the Sample A alloy and Sample B alloy increases accordingly, resulting in the decrease in magnetic properties [18]. Since Co is the γ-phase producer and enhancer for the same thermal treatments and composition, it favors the paramagnetic phase.

### 3.2. Optical Microscope (OM) Analysis

**Figure 4:**
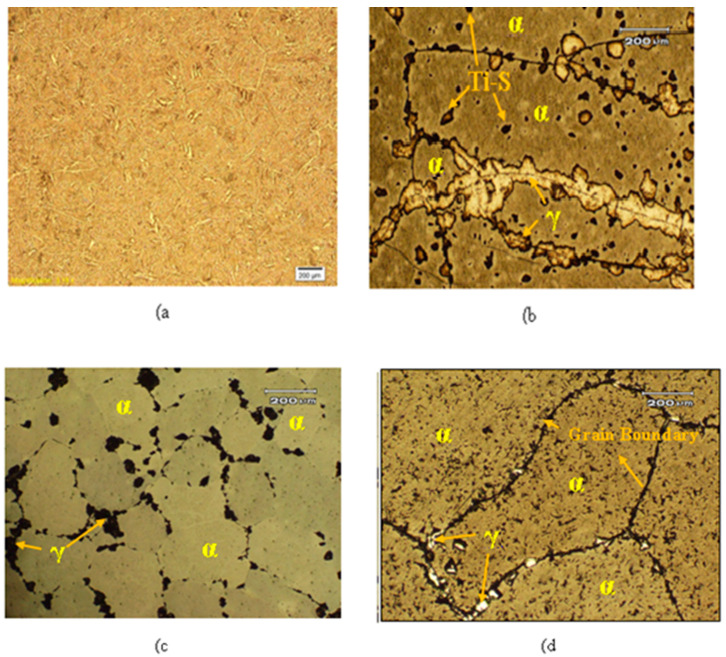
Optical micrograph consisting of single-phase alpha and gamma phases (**a**) the cast dendritic structure of (**b**) Sample A after complete heat treatment; (**c**) Sample B after complete heat treatment; and (**d**) Sample C after complete heat treatment.

In order to obtain the single solid solution phase, Sample A alloy, Sample B alloy and Sample C alloy were generally heat treated according to the manner shown in Figure 4. To obtain optimum microstructure and magnetic properties, the specimens were solution annealed at 1250 °C for 5 h and then rapidly quenched in compressed blowing air to capture high temperature single α-phase microstructure at room temperature. The rapid quenching is the desirable process to prevent undesirable allotropic phases starting, decrease the residual stresses, produce the crystal structure α-solid solution and complete the maximum diffusion for the homogenous polycrystalline structure.

The crystal structure obtained after solution annealing was bcc at room temperature which is compared with the microstructures of the cast and compressed air quenched after the homogenization of Samples A, Sample B and Sample C in Figure 4. In Figure 4a, grains with dendrites originate from the grain boundaries and spread out towards the center of the grain. These dendrites are produced under isotropic conditions, i.e., in the cast structure of the grain which later affects the magnetic properties. Since our requirement is the coarse grain or single phase structure, we solutionized the alloy at 1250 °C for 5 h. The micrographs in Figure 4b–d indicate single-phase α with a bcc crystal structure which was well confirmed by XRD analysis. The normal (average) grain size determined by linear intercept method in (40 + x)Fe–28Cr–(26 − x)Co–3Mo–1Ti–2V alloy was 300 ± 5 μm. Brittleness and the unwanted γ-phase increase with the increase in Co concentration in the investigated alloy, as indicated in the micrograph of the 24 and 26 Co content (i.e., Figure 4b,c). These unwanted phases enriched in Fe, confirmed by EDX analysis, deteriorates the magnetic properties in the alloys. The grain size indicates that vanadium is responsible for extending the FM α-phase which refines and prolongs the annealed microstructure [19]. Grain formation began at high temperature solidification due to nucleation concentrated towards the center throughout the specimens. These grains were separated from each other by the grains boundary, as shown in Figure 4d. Reduction in the properties is enabled by the carbon and nitrogen content present in the atmosphere or raw material which are gamma phase-forming material: therefore, carbon or nitrogen contained in the studied alloy extends the γ-phase region. The vanadium content in the alloy also suppressed the Ti carbides and nitrides, resulting in the expansion of the α-phase structure, which remains the same after the final step aging state. The EDX analysis confirmed the black spot within the grain as a Ti–S inclusion, as shown in Figure 4.

### 3.3. Microstructural Properties

**Figure 5:**
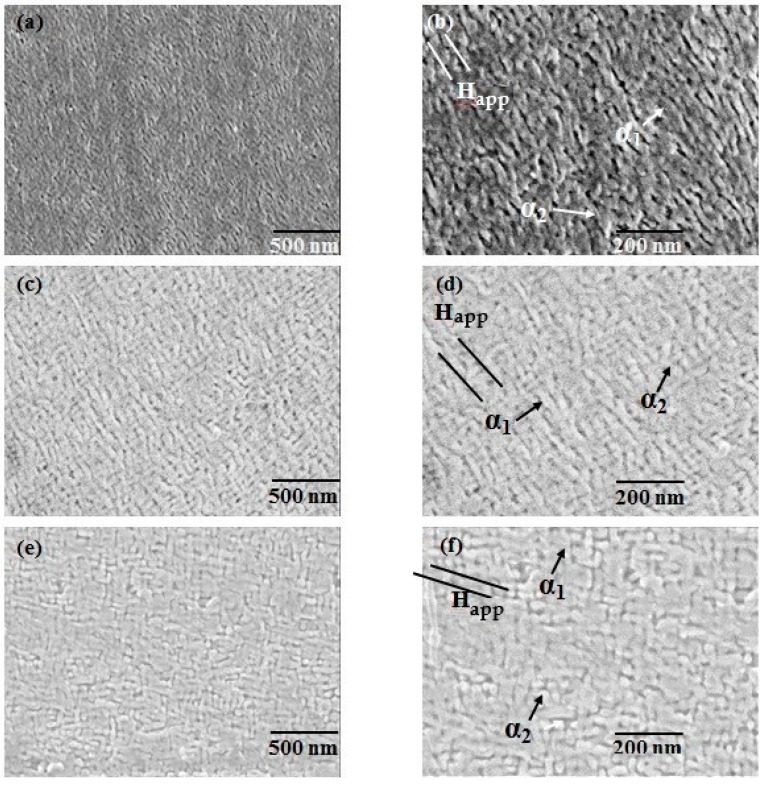
Field emission scanning electron microstructure showing spinodal phases with an α_1_-FM rod-like shape in the direction of the applied field embedded in the matrix of the α_2_-paramagnetic phase after complete heat treatment conditions: (**a**) Sample A; (**b**) amplified image of Sample A; (**c**) Sample B; (**d**) amplified image of Sample B; (**e**) Sample C; and (**f**) amplified image of Sample C.

**Figure 6:**
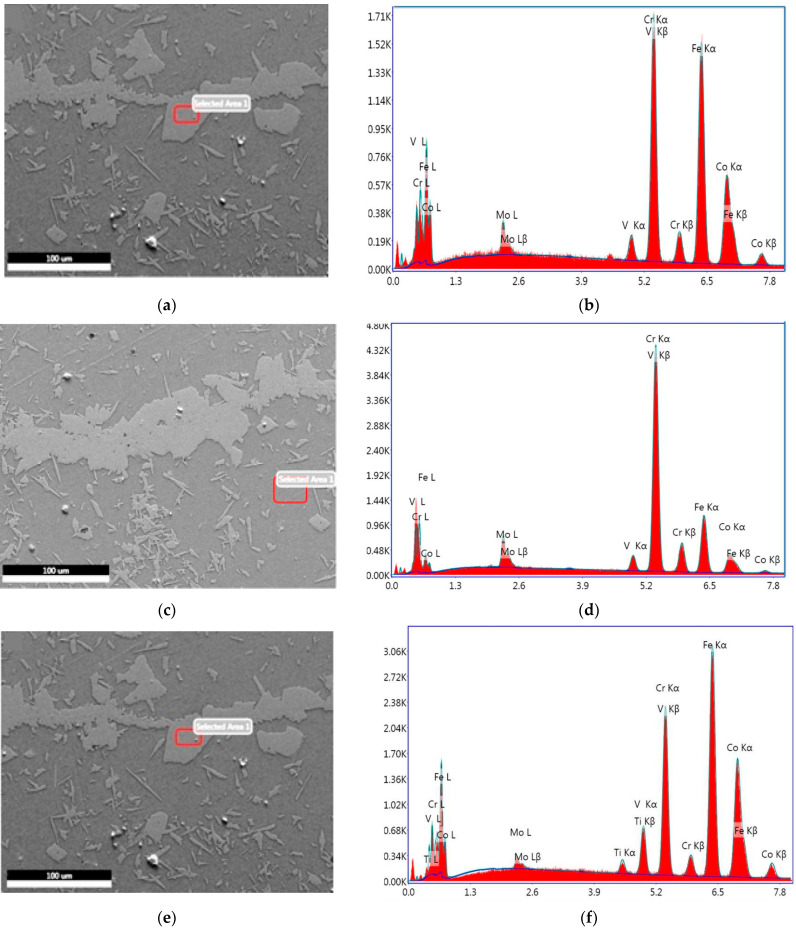
Energy dispersive X-ray analysis of selected area images and their cross ponding elemental composition spectra of (**a**) Sample A; (**b**) Spectrum A; (**c**) Sample B; (**d**) Spectrum B; (**e**) Sample C; and (**f**) Spectrum C.

The field emission scanning electron microscope (FESEM) microstructural analyses of the Sample A alloy, Sample B alloy and Sample C alloy are shown in Figure 5, while their cross ponding EDX micrographs and analysis spectra are shown in Figure 6. When the solution-annealed sample is subjected to thermomagnetic treatment, the α-phase is spinodally decomposed into α_1_ and α_2_ phases, where α_1_ is highly magnetic, i.e., an Fe, Co, Ti and V-rich phase and the α_2_-phase is weakly magnetic, i.e., Cr, Mo rich matrix. Both the Fe–Co α_1_-phase single-domain particles and Cr-rich α_2_-phase matrix have an isomorphic bcc structure. It was supposed that the phase parting α → α_1_ + α_2_ upon thermo-magnetic annealing at a temperature within the miscibility gap proceeds by spinodal decomposition. The α_1_ particles are of the single-domain size and rod-like shape, surrounded by weakly nonmagnetic α_2_ matrix [20]. It is speculated that in the TMT state, α_1_-particles which are supposed to precipitate at a temperature which is relatively high (620–640 °C) receive enough diffusion time and as a result, α_1_-particles grow and elongate in the applied field direction. This indicates that the number density, shape and elongation of α_1_-phase particles are critical for attaining noble magnetic properties in the alloys [21].

It was notable that the solution-annealed samples were nearly free from unwanted γ-phases which mostly appear on the grain borderlines. The dark spot in the grain was detected and recognized as nonmetallic Ti-rich presence areas which were confirmed by literature and FESEM analysis [22]. Basically, the field emission scanning electron microscope was not used for phase analysis because the electron beam began propagation at a high magnification and low voltage. It was simply shown that there are phases present in the sample. From the literature review, one can say which particle is α_1_ and α_2_. Figure 5 shows the spinodal decomposed microstructure after the multi-step aging state of Sample A alloy, Sample B alloy and Sample C alloy. The white rod-like single domain particles in the micrograph were probably embedded in the black α_2_-matrix as α_1_-phase which is in good agreement with the literature [23]. The phase line drawn on the phases gives an α_1_-phase composed of an Fe, Co, Ti, V and α_2_-phase which is mainly concentrated on Cr, Mo. In the micrograph, the α_1_ particle seems to be elongated along the direction of an applied magnetic field (marked with arrow). The degree of elongation is better in the 44Fe–28Cr–22Co–3Mo–1Ti which indicates that the V addition to the Fe–Cr–Co–Mo–Ti alloy facilitates the aligning and elongating of the Fe, Co-rich phase. The elongation of α_1_ improves the shape anisotropy which is responsible for the enhancement of the magnetic properties.

Figure 6 shows the energy-dispersive X-ray micrograph of the samples which clearly revealed that the white region is α_1_ and dark region is the matrix of α_2_-phase. The α_1_-phase embedded in the matrix of α_2_-phase.

### 3.4. Ferromagnetic Properties

Specimen ingots in cast shape have a high-temperature bcc α-phase solid solution with undesirable allotropic phases. The homogeneity and single phase of the specimens at room temperature required solution treatment followed by compressed blowing air quenching. The processing of the specimens contains the solution treatment, isothermal field treatment which was performed at 1250 °C for five hours, 630 °C for 30 min and subsequent aging at predetermined cooling rates. The temperatures, soaking times and subsequent aging with predetermined cooling rates were optimized via experimentations. A processing temperature exceeding or lowered from the optimized level produced surface degradation and undesirable allotropic phases. These unwanted phases then caused cracks in the quenched level as well as affected the microstructure of the specimens.

**Figure 7:**
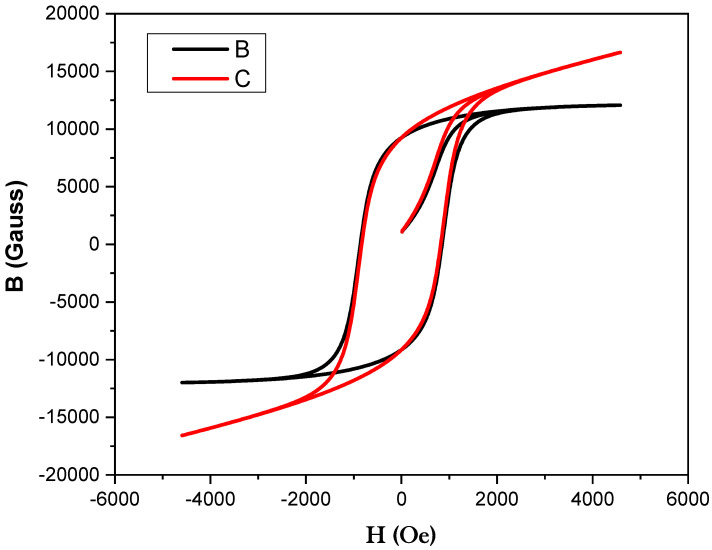
Magnetic hysteresis loop of Sample B and Sample C after complete treatment.

**Figure 8:**
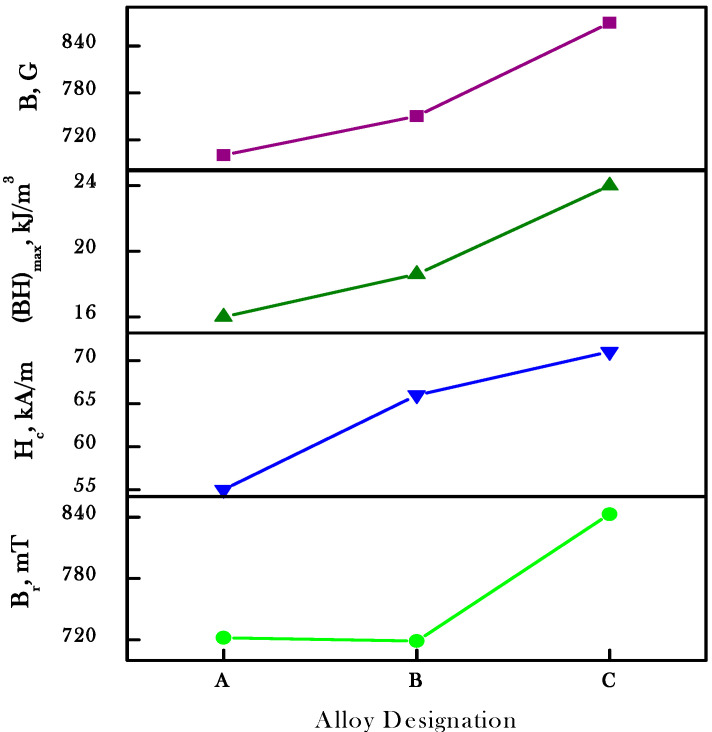
Magnetic properties vs. alloy designation of Sample A, Sample B and Sample C after complete treatments.

The best microstructure sample induced good magnetic properties in the alloys. The soaking time and magnetic field intensity were optimized for the isothermal field treatment (IFT) through experimentations which are 4.86 kOe and 30 min, respectively. The thermo-magnetic treatment are performed in the temperature range of 620–645 °C. The results revealed that magnetic properties strongly depend on the choice of TMT temperature. The difference in magnetic properties is related to the difference in elongation and volume fraction of spinodal phases produced during IFT and refined in aging treatments. Therefore, the optimum volume fraction of the spinodal phases was produced at 630 °C; thus, this was considered as the spinodal temperature for the studied alloys. The magnetic properties for Sample B alloy and Sample C alloy after complete treatment are presented in Figure 7, while their magnetic property parameters are depicted in Table 2. Figure 7 shows that the isothermal field treatment has a small effect on the 24Co (alloy B) than the 22Co (alloy C), meaning that the same elongation in the phases occurs at the optimized TMT temperature. The influence of the IFT temperature on alloy C due to the optimum level was achieved. The magnetic properties are increased with the increase in cobalt content, thus the optimum level are achieved for the ridge alloy 44Fe–28Cr–22Co–3Mo–1Ti–2V. The best magnetic properties of alloy C were B_r_ = 843 mT, H_c_ = 71 kA/m and (BH)_max_ = 24 kJ/m^3^. The addition of the cobalt content may contribute to the refining of the microstructure of the FM phase which enhances the magnetic properties. Magnetic properties versus the alloy designation after the complete heat treatment are shown in Figure 8, which contributed the better magnetic properties of the investigated alloy system.

Earlier literature emphasized the magnetic properties in high Co-containing Fe–(26–37) Cr–(16–25) Co alloys. Later, the studies shifted to low cobalt-containing Fe–(22–37) Cr–(6–15) Co alloys, as Co is expensive as well as strategic. Studies revealed that the high Co content in the Fe–Cr–Co alloys produced nonmagnetic gamma and sigma phases as indicated by optical microstructure properties, which is in agreement with the literature. Studies revealed that the gamma and sigma phases can be suppressed by the micro-addition of Mo, Ti or Nb elements. Mo addition to the ternary Fe–(22–30)Cr–(15–18.5)Co alloys is found to increase the magnetic properties due to aligning the spinodal phases α_1_ and α_2_ along the <100> directions. This means that good magnetic properties with Fe–Cr–Co–Mo alloys can be obtained by aligning and elongating the spinodal phases in <100> texture samples. The molybdenum content above 3.5 wt.% produced cracking and brittleness in the alloys due to the formation of the unwanted σ phase. The results indicate that the molybdenum content from 1 to 3.5 wt.% extends the FM α grain structure at the annealed state and affects the particle size, amount and shape anisotropy of the strongly magnetic α_1_ phase that in turn improves the magnetic properties of the ternary Fe–Cr–Co–Mo alloys [24,25,26,27].

## 4. Conclusions

From the investigation of the thermal, structural, microstructural and magnetic properties during solutionizing, the IFT and the subsequent aging treatment at predetermined cooling rates, the following conclusions can be made:

(i) The structure of the as-cast condition of a different composition consists of dendritic growing towards the center of the grain, reflecting low magnetic properties ranging from 50 to 60 Gauss.
(ii) Solutionizing samples retain the single-phase α solid solution from unwanted γ or δ phases depends upon the quenching condition and Co concentration.
(iii) Isothermal field treatment decomposes the single phase of alloys into two isomorphic phases and prolonged the FM phase in the applied field direction which then increases the magnetic properties.
(iv) IFT also increases the thermal stability, structural properties and refines the microstructure of the alloys.
(v) Step aging treatment provides the adjustment of composition due to which the volume fraction, difference in FM and paramagnetic phases increase and the microstructure is refined.
(vi) Co concentration enhances the magnetic properties and best magnetic properties achieved for alloy 44Fe–28Cr–22Co–3Mo–1Ti–2V are B_r_ = 843 mT, H_c_ = 71 kA/m and (BH)_max_ = 24 kJ/m^3^.
(vii) Except alloy C, the formed α solid solution single phase in Sample A alloy and Sample B alloy are not stable and can be transformed into the γ or δ phase during thermal treatments.
(viii) Because of the high thermal stability of the α phase in addition to good magnetic properties, it can be suggested that the 44Fe–28Cr–22Co–3Mo–1Ti–2V alloy is the best choice for the preparation of Fe–Cr–Co magnets by means of casting technique.

## Figures and Tables

**Table 1:** Chemical composition analysis of the alloys: Sample A, Sample B and Sample C.

Alloy ID	Fe	Cr	Co	Mo	Ti	V	C	S	N
**Sample A**	**40**	**28**	**26**	**3**	**1**	**2.0**	**0.004**	**0.012**	**0.020**
**Sample B**	**42**	**28**	**24**	**3**	**1**	**2.0**	**0.002**	**0.010**	**0.017**
Sample C	44	28	22	3	1	2.0	0.001	0.010	0.015

**Table 2:** Magnetic properties of the studied Sample A alloy, Sample B alloy and Sample C alloy after complete treatment investigating the alloy system.

Sample ID	Magnetic Properties			
	Flux Density, G	B_r_, mT	H_c_, kA/m	(BH)_max_, kJ/m^3^
A	700 ± 20	722	55	16
B	850 ± 20	819	68	22.6
C	870 ± 20	843	71	24

## Data Availability

The data presented in this study are available on request from the corresponding authors.

## References

[1] Ushakova O., Dinislamova E., Gorshenkov M., Zhukov D. (2014). Structure and magnetic properties of Fe–Cr–Co nanocrystalline alloys for permanent magnets. J. Alloys Compd..

[2] Kaneko H., Homma M., Nakamura K. (1972). New Ductile Permanent Magnet of Fe-Cr-Co System. AIP Conf. Proc..

[3] Ghasemi E., Ghasemi A., Hadi M., Sadeghi M., Hashemi S.H., Tavoosi M., Gordani G.R. (2018). Effect of cobalt doping on structural and magnetic characterization of nanocrystalline Fe72− xCoxCr28 (10 < X < 22) alloys. J. Magn. Magn. Mater..

[4] Shatsov A., Ryaposov I., Kozvonin V. (2017). Concertation-Inhomogeneous Hard Magnetic Alloys of the Fe–Cr–Co System with Elevated Content of Cobalt and Boron. Met. Sci. Heat Treat..

[5] Wu X., Bu S.-J., Han X.-H., Zhang C., Sun J.-B., Zhang Y., Pan Y.-F. (2016). Effects of Si and/or Ti Addition on the Microstructure and Magnetic Properties of Fe–Cr–Co Ribbons. IEEE Trans. Magn..

[6] Zhang L. (2016). Coercivity Enhancement and Gamma Phase Avoidance of Alnico Alloys. Master’s Thesis.

[7] Rehman S.U., Ahmad Z., ul Haq A., Akhtar S. (2017). Effects of Zr alloying on the microstructure and magnetic properties of Alnico permanent magnets. J. Magn. Magn. Mater..

[8] Rehman S.U., Jiang Q., Ge Q., Lei W., Zhang L., Zeng Q., Ul Haq A., Liu R., Zhong Z. (2018). Microstructure and magnetic properties of alnico permanent magnetic alloys with Zr-B additives. J. Magn. Magn. Mater..

[9] Souza C.A.C.d., Bolfarini C., Botta W.J., Lima L.R.P.D.A., Oliveira M.F.D., Kiminami C.S. (2013). Corrosion resistance and glass forming ability of Fe_47_Co_7_Cr_15_M_9_Si_5_B_15_Y_2_ (M = Mo, Nb) amorphous alloys. Mater. Res..

[10] Akbar S., Awan M.S., Aleem M.A., Sarwar M.N. (2014). Development of Mo containing Fe-Cr-Co permanent magnets by modified single step thermomagnetic treatment. IEEE Trans. Magn..

[11] Homma M., Horikoshi E., Minowa T., Okada M. (1980). High-energy Fe-Cr-Co permanent magnets with (BH) max≃ 8–10 MG Oe. Appl. Phys. Lett..

[12] Homma M., Okada M., Minowa T., Horikoshi E. (1981). Fe-Cr-Co permanent magnet alloys heat-treated in the ridge region of the miscibility gap. IEEE Trans. Magn..

[13] Sugimoto S., Satoh H., Okada M., Homma M. (1991). The development of <100> texture in Fe-Cr-Co-Mo permanent magnet alloys. IEEE Trans. Magn..

[14] Ahmad Z., ul Haq A., Yan M., Iqbal Z. (2012). Evolution of phase, texture, microstructure and magnetic properties of Fe–Cr–Co–Mo–Ti permanent magnets. J. Magn. Magn. Mater..

[15] Ahmad Z.u., Ul Haq A., Husain S., Abbas T. (2002). Magnetic properties of isotropic Fe–28Cr–15Co–3.5 Mo permanent magnets with additives. Phys. B Condens. Matter.

[16] Marín P., Aragón A., Escorial A.G., Lieblich M., Crespo P., Hernando A. (2013). Coercivity and its thermal dependence in microsized magnetic particles: Influence of grain boundaries. J. Appl. Phys..

[17] Menushenkov V.P., Shubakov V.S. (2015). Effect of secondary decomposition on coercivity of Fe-Co-Cr alloys with 15% Co. Solid State Phenomena.

[18] Korneva A., Korznikova G., Kashaev R., Sztwiertnia K. (2013). Microstructure of Hard Magnetic FeCr22Co15 Alloy Subjected to Tension Combined with Torsion at High Temperatures. Solid State Phenomena.

[19] Ustyukhin A., Ankudinov A., Zelenskii V., Milyaev I., Alymov M. (2017). Improvement of magnetic properties by hot rolling of sintered powder alloy in the Fe–Cr–Co system. Doklady Physical Chemistry.

[20] Vedmid L., Dorogina G. (2018). Iron–Chromium Precursors for Hard-Magnetic Fe–Cr–Co Alloys. Russ. Metall. (Met.).

[21] Rastabi R.A., Ghasemi A., Tavoosi M., Sodaee T. (2016). Magnetic characterization of nanocrystalline Fe80− xCrxCo20 (15 ≤ x ≤ 35) alloys during milling and subsequent annealing. J. Magn. Magn. Mater..

[22] Singh S., Wanderka N., Kiefer K., Siemensmeyer K., Banhart J. (2011). Effect of decomposition of the Cr–Fe–Co rich phase of AlCoCrCuFeNi high entropy alloy on magnetic properties. Ultramicroscopy.

[23] Shubakov V. (2013). Heat Treatment and Structure of High-Coercivity Alloys Based on the Fe–Co–Cr and Fe–Co–Cr–Mo Systems. Met. Sci. Heat Treat..

[24] Mukhamedov B., Ponomareva A., Abrikosov I. (2017). Spinodal decomposition in ternary Fe-Cr-Co system. J. Alloys Compd..

[25] Tao S., Ahmad Z., Khan I.U., Zhang P., Zheng X. (2019). Phase, microstructure and magnetic properties of 45.5 Fe-28Cr-20Co-3Mo-1.5 Ti-2Nb permanent magnet. J. Magn. Magn. Mater..

[26] Milyaev I., Yusupov V., Ostanin S.Y., Stelmashok S., Milyaev A., Laysheva N. (2018). Magnetic hysteresis and mechanical properties of hard magnetic Fe–27Cr–15Co–2Mo–Si–Ti–V alloy. Inorg. Mater. Appl. Res..

[27] Shubakov V. (2009). High-coercivity decomposition in Fe-(15, 23)% Co-30% Cr-3% Mo-0.5% Ti alloys. Russ. Metall. (Met.).

